# Simple Selection Procedure to Distinguish between Static and Flexible Loops

**DOI:** 10.3390/ijms21072293

**Published:** 2020-03-26

**Authors:** Karolina Mitusińska, Tomasz Skalski, Artur Góra

**Affiliations:** 1 Tunneling Group, Biotechnology Centre, Silesian University of Technology, ul. Krzywoustego 8, 44-100 Gliwice, Poland; k.mitusinska@tunnelinggroup.pl; 2 Biotechnology Centre, Silesian University of Technology, ul. Krzywoustego 8, 44-100 Gliwice, Poland; tomasz.skalski1967@gmail.com

**Keywords:** structure prediction, loop reconstruction, flexible loop, static loop, protein structure

## Abstract

Loops are the most variable and unorganized elements of the secondary structure of proteins. Their ability to shift their shape can play a role in the binding of small ligands, enzymatic catalysis, or protein–protein interactions. Due to the loop flexibility, the positions of their residues in solved structures show the largest B-factors, or in a worst-case scenario can be unknown. Based on the loops’ movements’ timeline, they can be divided into slow (static) and fast (flexible). Although most of the loops that are missing in experimental structures belong to the flexible loops group, the computational tools for loop reconstruction use a set of static loop conformations to predict the missing part of the structure and evaluate the model. We believe that these two loop types can adopt different conformations and that using scoring functions appropriate for static loops is not sufficient for flexible loops. We showed that common model evaluation methods, are insufficient in the case of flexible solvent-exposed loops. Instead, we recommend using the potential energy to evaluate such loop models. We provide a novel model selection method based on a set of geometrical parameters to distinguish between flexible and static loops without the use of molecular dynamics simulations. We have also pointed out the importance of water network and interactions with the solvent for the flexible loop modeling.

## 1. Introduction

Loops are the least organized elements among proteins’ secondary structures. On average, more than half of protein residues are located on loops [1]. The primary role of loops is to connect the more organized α-helices and β-strands, which allows the proteins to adapt their shapes. Therefore, loops are also linkers between domains in multi-domain proteins [2] and contribute to protein stability by the regulation of the folding process. They are mainly responsible for the dynamics of proteins’ shapes, and changes in their physico-chemical properties [3]. Loops can be involved in the proteins’ catalytic mechanism as regulatory elements [4], where they maintain the functional specificity of the protein by mediating biological processes such as the binding of small molecules and/or DNA, and/or access to the active site [4]. Loops are also found on the protein’s surface, where they are involved in protein–protein interaction, site recognition, signaling cascades, ligand and/or DNA binding, and catalysis [5]. Particular regions within proteins may remain flexible and unstructured due to its function, those are so-called intrinsically disordered proteins, which are highly abundant in nature [6].

Loops located on the protein’s surface, which are flexible and more solvent-exposed than other secondary structures, are especially problematic to characterize through experimental techniques such as CryoEM or X-ray crystallography. On the other hand, it was shown that nuclear magnetic resonance (NMR) could be helpful in determining the 3D structures of flexible loops, such as in Tim21 [7], and intrinsically disordered small proteins, such as amyloid-β fibrils [8]. Loop mobility introduces significant disorder: about 69% of the structures deposited in the Protein Data Bank (PDB) [9,10] are incomplete, and most often the loop regions are missing. The lack of part of the structural information of the experimentally solved structure might be related to the low resolution of the obtained structure and/or the flexibility of that particular region [11]. Based on the timescales of their dynamics, loops can be divided into slow (static, structural) and fast (dynamic, flexible, solvent-exposed) [2]. Fast loops have a relatively flat energy surface when compared to the thermal energy unit (k_B_T) which can be easily reshaped in the presence of a particular ligand. On the other hand, slow loops exhibit a higher energy barrier between different substrates, which may be difficult to reshape by introducing another substrate [2].

All types of loop are also susceptible to amino acid insertions and deletions, and are therefore considered as less conserved secondary structures. This means that homologous proteins are mostly different in their loop regions [12]. Due to the fact that the correct conformation of a given fragment has to be predicted based mainly on its sequence, loop reconstruction is a mini protein folding problem. Loops are generally too short to provide information about their local fold [13]. The missing residues may be reconstructed using protein structure prediction methods, such as homology modeling [14,15] or *Ab initio* modeling [16]. Homology modeling is one of the template-based structure prediction methods that use an experimentally solved structure of a close homologous protein as a template to predict the structure of the target protein. *Ab initio* modeling is based only on the physico-chemical information provided by the protein sequence. Among loop modeling software using homology modeling or *Ab initio* methods, a few are available as web servers, e.g., GalaxyLoopPS2 [17], DaReUs-Loop [18], LoopIng [19], Sphinx [20], and RCD+ [21], while some are available as standalone tools, such as MODELLER [22] and Rosetta-NGK (Next-generation KIC) [23]. A paper by DaReUs-Loop’s developers gives a brief comparison of the accuracy of these methods [18].

The aim of structure prediction software is to provide the most accurate models. Therefore, the method used to discriminate between native-like conformations and a set of decoys (non-native-like conformations) is as important as the model building technique itself. Most of the presented software use energy functions to point out the “best” models. RCD+ [21] uses an ICOSA energy function based on a pairwise coarse-grained contact potential of the inter-residue distance and orientation, LoopIng scores models based on a confidence function, and MODELLER uses the DOPE (Discrete Optimized Protein Energy) score [24], a pseudo energy score which is a sum of many terms, including some terms from the CHARM22 molecular dynamics force field, with spatial restraints based on the distribution of distances and dihedral angles in known protein structures [25]. SOAP-Loop and SOAP-PP (Statistically Optimized Atomic Potentials) [26] are also part of MODELLER; the SOAP potentials are attributed to the scoring of the orientation instead of distance, as well as the use of the recovery functions instead of a reference state.

To assess the accuracy of loop modeling and to evaluate the ability of the scoring function to select the “best” models, most of the software [27,28,29,30] use a dataset, such as Loops In Proteins (LIP) [31], containing experimentally solved proteins’ structures or loop regions as benchmark for their prediction method. Such an approach provides a convenient metric to compare the software with each other, although it says nothing about the accuracy of the prediction of loops without experimentally solved structures. Also, by using a dataset of already solved structures, the structure prediction software might be constantly improved towards less flexible, static loops [32]. Therefore, we hypothesize that current structure prediction software provide more accurate models of static loops, but might give inaccurate prediction of flexible solvent-exposed loops’ conformations making them similar to the static loops’ conformations [32]. Since the coordinates of flexible loops are often missing in the crystal structure, we believe that it is a quite common approach to reconstruct a flexible loop with a static loop template. In this paper, we consider the method for the selection of accurate loop models of static and flexible loops and their accuracy examination using classical molecular dynamics (MD) simulations. We propose a geometry-based selection method to enhance conformational sampling of the obtained loop models. By combining a set of geometrical parameters and model quality assessment methods from the MODELLER software (DOPE and SOAP scores), we achieved more detailed structural information of the loops structure. Using such information, we were able to search for different conformations of reconstructed loops and therefore extend the sampling. We chose two epoxide hydrolases belonging to a large α/β-hydrolase family as a case study structures, due to their relatively small size and the lack of a flexible loop corresponding to a static loop in the second structure, which is probably the most common situation in loop reconstruction.

We are going to check whether we were able to improve the examination of the loop models accuracy just by extending the simulation length up to 500 ns and compare these results with the results from a set of shorter simulations.

## 2. Results

**Figure 1:**
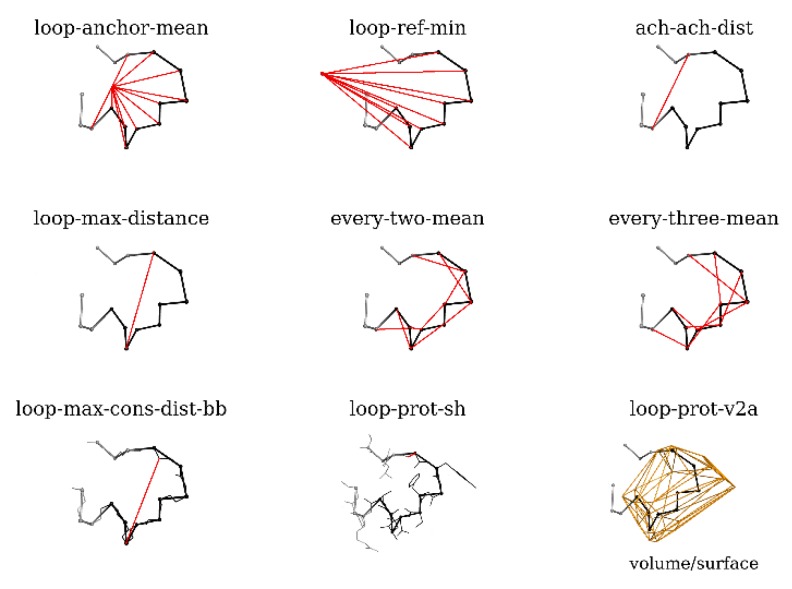
The set of nine geometry-based loop parameters (see *Methods* section for details). The analyzed loop is shown as black lines, with Cα atoms marked as small balls. The red lines show the distances used for calculations; the orange shape represents the ConvexHull approximation of the volume of loop atoms.

The proteins used in this case study belong to the α/β-hydrolases superfamily and therefore share the same structural pattern: the conservative core domain consists of eight superhelically twisted β-strands with α-helices at both sides, covered by a mostly helical variable cap domain [33]. The active site is buried between the core and the cap domains. The *Aspergillus niger* epoxide hydrolase (AnEH) structure has a gap of a missing nine-amino-acid-long loop located at the border between the cap and the core domain which was selected as a representative FL (flexible loop). We assumed that the missing loop of AnEH could be a flexible loop due to i) its localization in proximity of the entrance to the active site, ii) the lack of coordinates in every available crystal structure, and iii) the work of Reetz [34,35] and Kotik [36] in which they have mutated residues near the active site and thus regulate the enzyme’s activity. The corresponding region of the *Bombyx mori* juvenile hormone epoxide hydrolase (BmJHEH) structure is not involved in the catalytic mechanism and is abundant in α-helices, which might suggest that it is a static loop. Also, the space surrounding the selected loop is smaller than in the case of the missing flexible loop of AnEH. The representative SL (static loop) in both structures is located between the β5 strand and the α5 helix of the α/β-hydrolase scaffold. The selected loop was manually removed from the BmJHEH structure, and the corresponding static loop of the AnEH was used as a template. According to [37,38], loops of that length are most likely to have a different conformation to that of the native protein. Both the SL and FL in this case study were reconstructed using the same loop modeling protocol, and models for further analysis were chosen using the model selection method described in the *Methods* section. In both cases, 10,000 loop models were built and described using geometry-based parameters (Figure 1, Figure S1; for parameters description see *Methods* section), combined with the DOPE and SOAP scores from the MODELLER software. 

**Figure 2:**
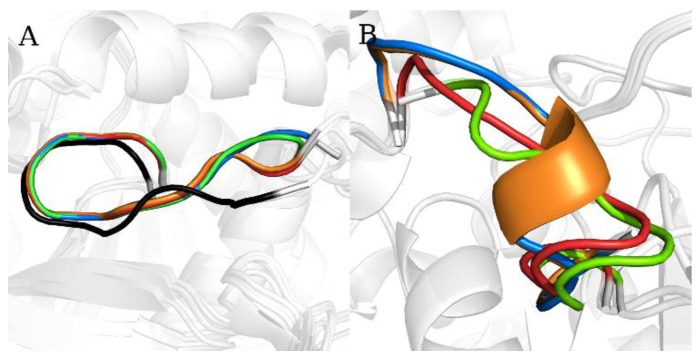
The loop models of reconstructed (**A**) static and (**B**) flexible loops. The SL_m1 and FL_m1 models are blue, SL_m2 and FL_m2 are red, SL_m3 and FL_m3 are green, SL_mDOPE and FL_mDOPE are orange, and the WT model of the SL is black. The protein is shown in cartoon representation and colored white.

We then preprocessed them using the WEKA software’s implementation of PCA and divided them into separate clusters, which gave us several models that were used as starting points for simulations; we referred to them as SL_m1, SL_m2, and SL_m3 for the models of the static loop, and as FL_m1, FL_m2, and FL_m3 for the models of the flexible loop (Figure 2). In the next sections, we will describe the accuracy of the chosen loop models using MD simulations of various lengths.

### 2.1. Static Loop Reconstruction

**Figure 3:**
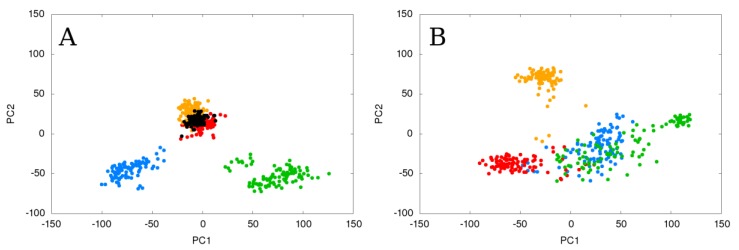
Normal modes analysis results of the (**A**) static loop (SL) and (**B**) flexible loop (FL) simulations using particular SL and FL models as starting points. The points representing SL_m1 and FL_m1 are blue, SL_m2 and FL_m2 are red, SL_m3 and FL_m3 are green, SL_mDOPE and FL_mDOPE are orange, and the wild-type of SL is black. Please note the spread of points on both graphs and that on the SL graph points representing simulations of three SL models are found on the same space (SL_m2, SL_mDOPE, and WT), while on the FL graph the points of three FL models overlap (FL_m1, FL_m2, and FL_m3) and one model is separated and less spread out (FL_mDOPE).

We conducted a single 100 ns MD run of each selected model to examine the accuracy of static loop models. The SL models were stable, the reconstructed loop remaining in the same position as it was at the beginning of the simulation. Since the crystal structure of the selected SL is known, we ran an additional 100 ns MD simulation and referred to it as a wild-type model (WT). One of the three proposed loop models (starting point model: SL_m2) had similar RMSD (Root Mean Square Deviation) and RMSF (Root Mean Square Fluctuation) values to the wild-type and to the model with the lowest DOPE score (starting point model: SL_mDOPE) (Figure S2). After more than 66 ns of the simulation, the SL_m2 model had slightly changed the analyzed loop’s conformation from about 1.04 Å on average to about 0.45 Å on average, which made it more similar to the wild-type model. The other SL models had higher RMSD values; in a simulation where the SL_m3 model was the starting point, the RMSD values reached the maximum at 3.21 Å. We analyzed the normal modes of the selected models using frames collected every 1 ns and observed that points representing simulations where the wild-type model, the model with the lowest DOPE score, and one of the proposed loop models (SL_m2) had been used as starting points, were all found in the same conformational space, while points representing two other simulations were found separately. The normal modes graph showed also that the SL models are focused in small groups, which suggests that each model adopts a particular conformation and does not switch to others (Figure 3A). By comparing the normal modes of the simulations of the SL models with the simulation of the wild-type structure, we were able to select the method of loop reconstruction which gave the model that was the most similar to the wild-type. We also averaged the coordinates of each model during the whole simulation run to search for the closest converged model. The structure of the model with the lowest DOPE score averaged along the simulation frames was the most similar to the averaged structure of the wild-type model (Table 1, Figure S4). These results are in agreement with the commonly used approach, and the selection of the model with the lowest DOPE score for the reconstruction of a static loop can be recommended. The high number of created loop models will only improve model quality.

### 2.2. Flexible Loop Reconstruction

Since the FL coordinates are unknown in the crystal structure, we are not able to compare our results with simulations of the wild-type model. Although in the PDB database three different crystal structures of AnEH are available, each of them has a gap of missing coordinates in the FL region. Therefore, we used an approach that is similar to the SL analysis, using the FL_mDOPE model as the reference point and the FL models gave significantly different results. Two of the loop models used as starting points (FL_m1 and FL_m3) underwent conformational changes in the reconstructed flexible loop region; during these simulations, the RMSD values reached the maximum at 3.83 and 3.06 Å, respectively. At about 68 ns of the simulation time, where the FL_m3 model was the starting point, we observed changes in its overall conformation from 2.06 up to 3.29 Å (Figures S3 and S5). The normal modes analysis showed that the three proposed loop models used as starting points for MD simulations (FL_m1, FL_m2, and FL_m3) were found in a different conformational space to the simulation of the model with the lowest DOPE score (FL_mDOPE). Also, the points representing the simulations of the proposed loop models were spread out on that space and overlapped with each other, whereas the simulation of the FL_mDOPE model was separated (Figure 3B). The analyzed FL is located at the border between the core and cap domains, not surrounded by other secondary structures, and therefore displays greater scope for conformational changes than the static loop.

### 2.3. Flexible and Static Loops’ Comparison 

**Figure 4:**
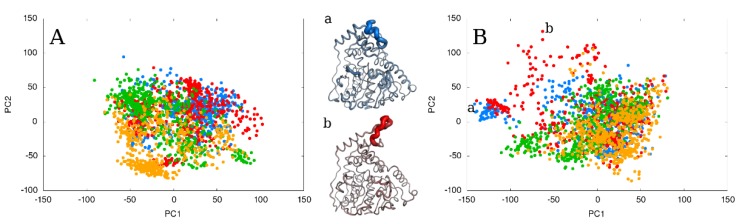
The results of normal modes analysis of five repetitions using particular FL models as a starting point for simulation during (**A**) 100 and (**B**) 500 ns MD runs. For picture clarity, only every 5th point on the right panel is shown. The points representing FL_m1 are blue, FL_m2 are red, FL_m3 are green, and FL_mDOPE are orange. The points representing each group of simulations with a particular FL model used as a starting point are widely spread and overlap. On the left panel, the points representing the FL_mDOPE model populate a region of the conformational space which is not visited by other models. On the right panel, the points representing the FL_m1 and FL_m2 models populate a region of the conformational space not visited by other models (shown as (**a**) (blue) and (**b**) (red)) which is related to movements of the N-terminal meander. The thickness of each structural element is related to its movements against the averaged structure.

During the analysis of the differences between the simulations of the FL and SL models, we found that the distribution of geometry-based parameters was significantly different. For the FL models, the parameters related with the loop’s arch-shape, such as loop-ref-min, loop-max-distance, ach-ach-dist and loop-anchor-mean, are widely distributed (RSD > 8%), whereas for the SL models, only the ach-ach-dist shows such high variance (RSD = 12.9%) (Table S1). The position of the SL loop is limited by the neighboring secondary structures and therefore the number of its possible conformation is also restricted. Hence, the main shape of the SL remains unchanged, and the greatest variability of the created models is between the anchoring residues (Figure 2A). We believe that this is a quite common situation for static loop reconstruction to fill the gap between other secondary structures of the protein. As for the FL model, the position of the loop is not restricted by adjacent secondary structures; the FL might be exposed towards the solvent or coiled near the protein surface or, as it is in our case, near the entrance to the active site. We have also found that the loop-ref-min, loop-max-distance, ach-ach-dist for the FL models, and ach-ach-dist parameters for the SL models, show statistically significant differences. The changes in the loop-anchor-mean parameter describe the ability of the loop to bend and adopt different conformations. Therefore, in the case of the SL reconstruction, we were able to select the “best” model on the basis of the PCA results; the points representing simulations where the SL_m2 and SL_mDOPE were used as starting points were overlapping with the simulation of the wild-type structure. Also, the points representing each simulation were less spread out (Figure 3). In the case of the FL reconstruction, where the structure of the wild-type was unknown, the PCA results were ambiguous, since the three simulations of particular FL models used as starting points overlapped, while one was separated and less spread out. The less spread out points represented the simulation of the FL model with the lowest DOPE score (FL_mDOPE), which was found in the same conformational space as the simulation of the wild-type structure of SL, possibly suggesting that the FL_mDOPE model represents static-like conformation rather than flexible-like (Figure 4). On the other hand, the points representing the simulations of the three other FL models (FL_m1, FL_m2, and FL_m3) were widely spread across the conformational space and overlapped with each other, which might suggest the possibility of conformational changes of the analyzed FL, which is also intuitively in agreement with the FL characteristics.

### 2.4. The Effect of Running Repetitions of Simulations 

**Figure 5:**
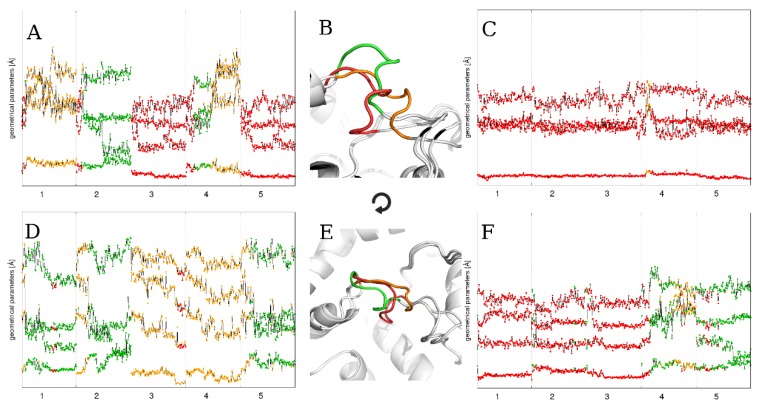
The time evolution of the four statistically significant loop parameters during five 100 ns MD runs using particular flexible loop models as a starting point: (**A**) FL_m1, (**C**) FL_m2, (**D**) FL_m3, and (**F**) FL_mDOPE, and (**B**) averaged loop structures in identified conformations: “open” (green), “semi-open” (orange), and “closed” (red), and (**E**) top view. The numbers on the horizontal axes represent the simulation repetition. Please note the colored points representing each loop conformational state and how the loop changes its conformation depending on the loop model used as a starting point.

Since the results of the flexible loop’s reconstruction were ambiguous, we decided to run four more repetitions of the simulations of each FL model (FL_m1, FL_m2, FL_m3, and FL_mDOPE) to enhance the conformational sampling. We obtained in total 2 µs of MD simulations (four models, five repetitions; 100 ns each). We still observed conformational changes in the reconstructed loop, but in the simulations of only two models - FL_m1 (in one run the RMSD value reached 3.83 Å) and FL_m3 (in one run the RMSD changed from 1.79 Å to 4.74 Å) - whereas simulations of the two other models, FL_m2 and FL_mDOPE, were stable (fluctuating around 2.0 Å) (Figures S6–S9). These observations are related to the shape of the reconstructed loop; during the simulations of the stable FL_m2 and FL_mDOPE, the loop was wrapped towards the active site, whereas during the simulations of the flexible models FL_m1 and FL_m3, the loop was more solvent-exposed. Such differences are shown on the normal modes graph, where points representing simulations of each FL model populate the same conformational space, but there are also regions where one of them prevails (Figure 4). However, the statistical analysis did not show similarities between each group of repetitions (Figure S10). To gain insights on the conformational states of the FL and possible transitions between them, we used four statistically significant geometrical parameters (loop-anchor-mean, loop-ref-min, ach-ach-dist, and loop-max-distance), which were employed for the loop’s shape description and clustered the states into groups representing the “open”, “closed”, and “semi-open” conformations (Figure 5, Table S3). We observed that dividing the data set into more clusters caused some overlap between the averaged loop conformations. The simulations of the FL_m2 model, which was also one of the most stable models, did not undergo any conformational changes and were mostly in the “closed” conformation, whereas during simulations of other models, switching between states occurred. Using more repetitions of the same starting point model enhanced conformational sampling and helped determine the loops’ states. We were also able to search for the most-populated loop state depending on the starting point model. The proposed model selection method provided models that represented three different conformations of the analyzed loop during simulations (FL_m1 - the “closed” and “semi-open” conformations, FL_m2 - the “closed” loop, and FL_m3 - the “open” loop), whereas the commonly used FL_mDOPE model remained in the “semi-open” position during the simulations and very rarely switched to other conformations (Table S2).

### 2.5. The Effect of Extending the Simulation Length 

**Figure 6:**
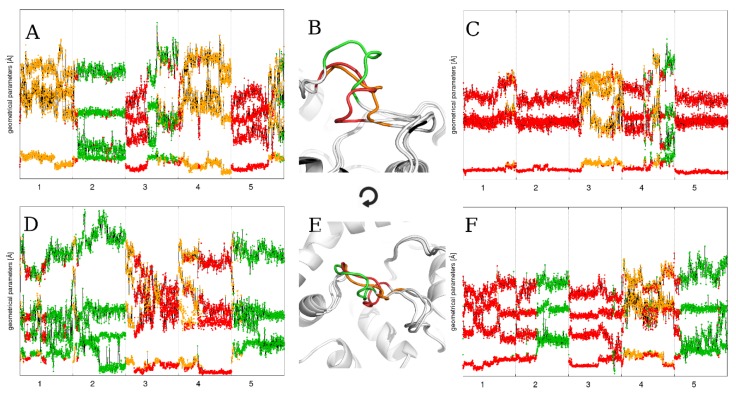
The time evolution of the four statistically significant loop parameters during five 500 ns MD runs using particular flexible loop models as a starting point: (**A**) FL_m1, (**C**) FL_m2, (**D**) FL_m3, and (**F**) FL_mDOPE, and (**B**) averaged loop structures in identified conformations: “open” (green), “semi-open” (orange), and “closed” (red), and (**E**) top view. The numbers on the horizontal axes represent the simulation repetition. Please note the colored points representing each loop conformational state and how the loop changes its conformation depending on the loop model used as a starting point. The loop structures representing each state are close to those from a similar analysis for 100 ns MD runs.

We extended each run to check whether we would be able to observe more transitions and conformational changes. By extending the simulations to a total of 10 µs (four models, five repetitions, 500 ns each) we did not ensure better conformational sampling. We still observed only one transition per simulation run, with only rarely switching back to the initial state (Figure 6, Table S3). The RMSD and RMSF plots gave the same level of information on loop behavior as the 100 ns MD simulations with five repetitions did (Figures S11–S14). Generalized linear models (GLM) analysis showed that the extension of the simulations did not improve the variability of the models (Table S4). We also observed that the conformational space populated during extended simulations shifted left to the top-left space of the PCA results graph. These conformational changes are related to the movements of the N-terminal meander region of the AnEH structure. Longer simulations of the FL_mDOPE model also slightly increased the sampling of the “open” loop conformation compared to shorter simulations (7.2% of “open” conformation during 100 ns MD runs and 32.4% of “open” conformation in 500 ns MDs) (Table S3).

### 2.6. Relationship between the Total Energy and DOPE Score 

**Figure 7:**
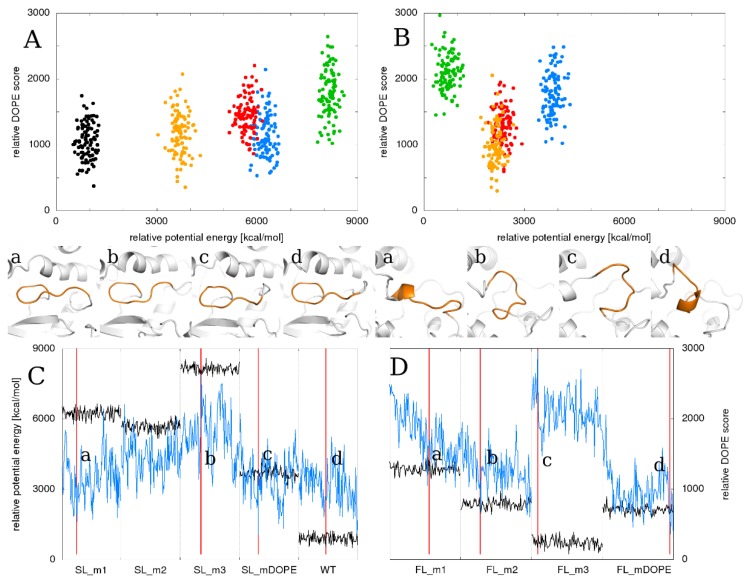
The relationship between the potential energy of the system, its DOPE (Discrete Optimized Protein Energy) score and the shape of its reconstructed SL using particular SL models (left side) and FL models (right side) as starting points for simulations. The potential energy (black lines) and DOPE score (blue lines) values are relative to show the distances between the models. The upper panel shows clusters of each (**A**) SL and (**B**) FL model in the context of their potential energy during MD simulation and the DOPE score of the loop model. The lower panel shows the shapes of the analyzed (**C**) SL and (**D**) FL models in particular simulations. It should be noted that the almost α-helically folded shape of the FL is the most favorable by DOPE score and at the same time the least favorable in terms of the potential energy of the system.

We decided to also check the relationships between the time evolution of the potential energy of each system and their DOPE score (Figure 7). In the case of the SL reconstruction, the values of the DOPE score were comparable between the simulations of wild-type and the loop model with the lowest DOPE score, SL_mDOPE. The values of the potential energy of this system were closer to the values observed in the simulations of other loop models than to the wild-type model. On the other hand, during the simulation of the SL_m3 model, which had the highest DOPE score and potential energy, the loop’s shape affected the shape of adjacent secondary structures (see the shape of the β-strand below the SL in Figure 7). In the case of the FL reconstruction, the results are quite different: during the simulation of the model with the lowest potential energy values, the DOPE score values were the highest while during the simulation of the model with the lowest DOPE score, FL_mDOPE, the potential energy values were second lowest. The most favorable FL conformation, according to the DOPE score, was when the loop was partially folded into an α-helix, whereas the potential energy favored the solvent-exposed shapes of the FL. Therefore, we recommend the potential energy as the model evaluation method of a solvent-exposed loop rather than the DOPE score.

## 3. Discussion

Protein structure prediction software is specialized towards the modeling of properly packed and compact structures. It provides models of high accuracy comparable with the experimentally solved structures. Many model evaluation methods have been developed to discriminate between decoys and native-like models, e.g., DOPE score in MODELLER, DFIRE [39], VERIFY3D [40], PROSA [41], and PROCHECK [42]. Although in the case of highly flexible intrinsically disordered regions within proteins [6], and solvent-exposed loops which could be related to the enzyme’s catalysis, substrate binding, or product release, these methods can be insufficient [32]. Liu et al. provided a comprehensive review on computational tools focused on the prediction of intrinsically disordered regions and proteins [43]. In the case of flexible loop reconstruction, there is no “best” model, but a number of “good” models, which might be used to observe the conformational changes of the reconstructed loop. We hypothesize that solvent-exposed loops are lowering their potential energy by interacting with water molecules in their surroundings. In a recently published study on the crystallographic structures of *Aspergillus usamii* E001 epoxide hydrolase (PDB IDs: 6ix2 and 6ix4), the reconstructed flexible loop was found in a conformation similar to the “open” state presented here (Figure S15). Also, such hypotheses that solutes could alter loops’ conformations have been supported by other papers, such as a work by Kim et al. [44,45] where they showed that bulky PEGs effectively block the substrate-induced conformational changes of vitamin B12 transporter, BtuB [45]. β-Lactoglobulin forms a water network to ensure an open state of a loop located near the active site. When the water network is disturbed, the loop adopts a partially closed conformation. The loop closes access to the active site at a pH below 7 [46]. We suggest that structure prediction software should include the water network effect on the loops in their predictions.

In this paper, we propose a novel approach towards loop model selection and evaluation for flexible, solvent-exposed loops, based on the statistically significant loop parameters, i.e., loop-anchor-mean, loop-ref-min, ach-ach-dist and loop-max-distance, describing the loop’s shape. We have also found that the distribution of geometry-based parameters is different in FL and SL models, giving us a simple method to distinguish between those two different loop types. The SL models have a wider distribution of the ach-ach-dist parameter related to the distance between loop anchoring residues. This suggests that the general shape of the SL remains the same, while the most diverse part is the anchoring residues. Although the distance between the anchoring residues might affect the accuracy of the loop prediction [32,47], we think that this is not the case here. The SL models retain most contacts with the surrounding residues and therefore remain in almost the same position in every 10,000 loop models. In the case of FL, all four parameters have shown great variability and we have found statistically significant differences in their distributions, hence we were able to identify several FL loop’s conformations without the use of molecular dynamics simulations. In our systems of interests, the FL is located at the border between the core and the cap domains, where it is distant from the other secondary structures and may be interacting with the solvent.

We have also shown that in the case of static loop reconstruction, the DOPE scoring method is efficient (when using a relatively large set of created models) and provides accurate models. On the other hand, we have shown that in the case of flexible loops, the DOPE score would favor partially folded structures rather than solvent-exposed conformations which could be adopted by flexible loops engaged in catalytic processes. Therefore, for the reconstruction of flexible, solvent-exposed loops, which are often missing from the crystal structures, we propose the use of the potential energy rather than DOPE score to evaluate the model. We have shown also how the loop reconstruction approach can be used to identify alternative flexible loop conformations.

We have also shown that extending the simulations’ length from 100 up to 500 ns does not significantly enhance the conformational sampling or provide better understanding of the transitions between the loop’s states. In contrast, we observed AnEH’s conformational changes related to the N-terminal region’s movements when we extended the simulations, in line with the study of [48]. We therefore recommend for MD simulations using more repetitions of sufficient length, rather than extending the simulations towards microsecond lengths. Longer unrestrained simulations might have a tendency to move away from the native-like structures [49].

## 4. Materials and Methods 

### 4.1. Loop Reconstruction 

The *Aspergillus niger* epoxide hydrolase (AnEH, PDB ID: 1qo7 [50]) and *Bombyx mori* juvenile hormone epoxide hydrolase (BmJHEH, PDB ID: 4qla [51]) structures were downloaded from the Protein Data Bank (PDB). Both AnEH and BmJHEH belong to the microsomal epoxide hydrolase sub-family, share the α/β-hydrolase scaffold [33], and their sequences are 29.4% identical, according to a BLAST search [52]. The structure of AnEH has a gap of missing coordinates representing a 9-amino-acid-long loop region, which represents the flexible loop. The other available crystal structures (PDB IDs: 3g02 and 3g0i [53]) are also missing such information. This AnEH region corresponds to the BmJHEH extended all-α cap domain region with residual flexibility only, which is the static loop template for the missing flexible loop. The BmJHEH structure is complete and there are no other isoforms available on the PDB database. For comparison, we have selected two corresponding loops representing static loops in both structures. They are located between the β5 strand and the α5 helix of the conserved α/β-hydrolase scaffold. In the latter part of this paper, we shall refer to loops involved in static loop reconstruction as SL and those used in flexible loop reconstruction as FL. The corresponding SL and FL were reconstructed using MODELLER 9.17 to build 10,000 homology models for each loop following the Basic homology modeling tutorial (https://salilab.org/modeller/tutorial/basic.html). Each homology model was optimized and then refined using molecular dynamics with simulated annealing (md_level=refine.very_slow). Figure S1 shows the diagram of the loop reconstruction and model selection method.

### 4.2. Model Selection

As the input data, the DOPE and SOAP scores obtained from MODELLER 9.17 were combined with nine geometrical parameters describing the loop’s shape (see Figure 1):

- loop-anchor-mean: mean distance from the Cα atoms of loop residues and the centroid of the Cα atoms of the loop attachment points (residues 188 and 196 in BmJHEH and residues 318 and 326 in AnEH),
- loop-ref-min: minimum distance from the Cα atoms of loop residues to the centroid of the Cα atoms of a conserved Trp residue located in the proximity of the active site in both structures (Trp154 in BmJHEH and Trp117 in AnEH),
- ach-ach-dist: distance between the Cα atoms of the attachment points,
- loop-max-distance: maximum distance between Cα atoms of the loop,
- every-two-mean: mean value of the distance between Cα atoms of every second residue of the loop,
- every-three-mean: mean value of the distance between Cα atoms of every third residue of the loop,
- loop-max-cons-distance-bb: maximum distance between the loop backbone atoms (Cα, C and N),
- loop-prot-sh: the minimum distance between the loop and protein (non-loop) atoms,
- loop-prot-v2a: measure of loop sphericity; a ConvexHull approximation of the volume to area ratio of the loop.

The calculated data were then preprocessed using WEKA software [54], using its implementation of principal component analysis (PCA) to reduce the variability of the geometry-based parameters. Clustering was then performed using a Simple K-means method to divide the preprocessed data into separate clusters. As a representative of each cluster, the model which was the median was chosen. The model with the lowest DOPE score, which represents the most commonly used approach for model selection, was chosen for comparison. As a result of the model selection method, median models for SL and FL were selected; we shall refer to them as SL_m1, SL_m2, and SL_m3 for models of the static loop, and as FL_m1, FL_m2, and FL_m3 for models of the flexible loop. The models with the lowest DOPE score are marked in the latter part of the paper as SL_mDOPE and FL_mDOPE for the static and flexible loop models, respectively.

### 4.3. Statistical Analysis of Loop Parameters

To model the relationship between the parameters and simulation lengths, we used generalized linear model analysis with a Poisson distribution and log link function for all measurements [55]. Only significant relationships between the parameters and models were chosen for further analyses. DOPE and SOAP scores were log model transformed to improve the performance of the models. Post hoc Tukey’s honestly significant difference (HSD) tests were used to determine the statistical significance of differences in mean parameters and simulation lengths (sim). Statistical analyses were performed using STATISTICA software [56].

### 4.4. Molecular Dynamics Simulations and Normal Modes Analysis

H++ server [57] was used to protonate each structure using standard parameters and pH 7.5. LEaP [58] was used to add counterions and immerse models in a truncated octahedral box of TIP3P water molecules. Water molecules were placed inside the protein using the combined methods of 3D-RISM [58] and the Placevent algorithm [59]. Amber 14 [58] was used to run one 100 ns simulation of BmJHEH wild-type and the selected models, and 500 ns simulations of AnEH loop models. The minimization procedure consisted of 2000 steps, involving the 1000 steepest descent steps, followed by 1000 steps of conjugate gradient energy minimization, with decreasing constraints on the protein backbone (500, 125, and 25 kcal·mol^−1^·Å^−2^) and a final minimization with no constraints on conjugate gradient energy minimization. Gradual heating was performed from 0 to 300 K over 20 ps using a Langevin thermostat with a temperature coupling constant of 1.0 ps in a constant volume periodic box. Equilibration and production were run using constant pressure periodic boundary conditions for 2 ns with a 1 fs time step and 100 ns with a 2 fs time step, respectively. A constant temperature was maintained using the weak-coupling algorithm for 100 ns of the production simulation time, with a temperature coupling constant of 1.0 ps. Long-range electrostatic interactions were modeled using the Particle Mesh Ewald method with a non-bonded cut-off of 10 Å and the SHAKE algorithm. The coordinates were saved at intervals of 1 ps. The normal modes analysis was conducted by CPPTRAJ [60] from the Amber18 suite, the coordinates being saved every 1 ns. 

## 5. Conclusions

Using the model selection method based on geometrical parameters presented in this paper, we were able to determine different loop conformations without using MD simulations. Such a model selection method is also useful for discrimination between static and flexible loops, since their shapes and overall behavior are different. We also pointed out the effect of the solvent on the loop’s conformations. The flexible, solvent-exposed loops might by lowering their potential energy by interacting with the water molecules from the solvent.

## Figures and Tables

**Table 1:** The RMSD (Root Mean Square Deviation) values of the averaged static loop (SL) structures compared with the averaged wild-type structure. For the RMSD calculations the mentioned atoms from the whole structure were used.

	SL_m1	SL_m2	SL_m3	SL_mDOPE
Heavy atoms RMSD [Å]	2.08	2.00	2.12	1.67
Cα atoms RMSD [Å]	1.76	1.63	1.73	1.34

## Supplementary Materials

Supplementary materials can be found at https://www.mdpi.com/1422-0067/21/7/2293/s1.

## Abbreviations

AnEH	*Aspergillus niger* epoxide hydrolase
BmJHEH	*Bombyx mori* juvenile hormone epoxide hydrolase
SL	Static loop
FL	Flexible loop
