# Supplementary material for: Simple Selection Procedure to Distinguish between Static and Flexible Loops
Source: Int J Mol Sci. 2020 Mar 26;21(7):2293. doi: 10.3390/ijms21072293 (PMC7177474; doi:10.3390/ijms21072293)
Supplement: Supplementary file 1 [file ijms-21-02293-s001.pdf]

Supplementary Materials

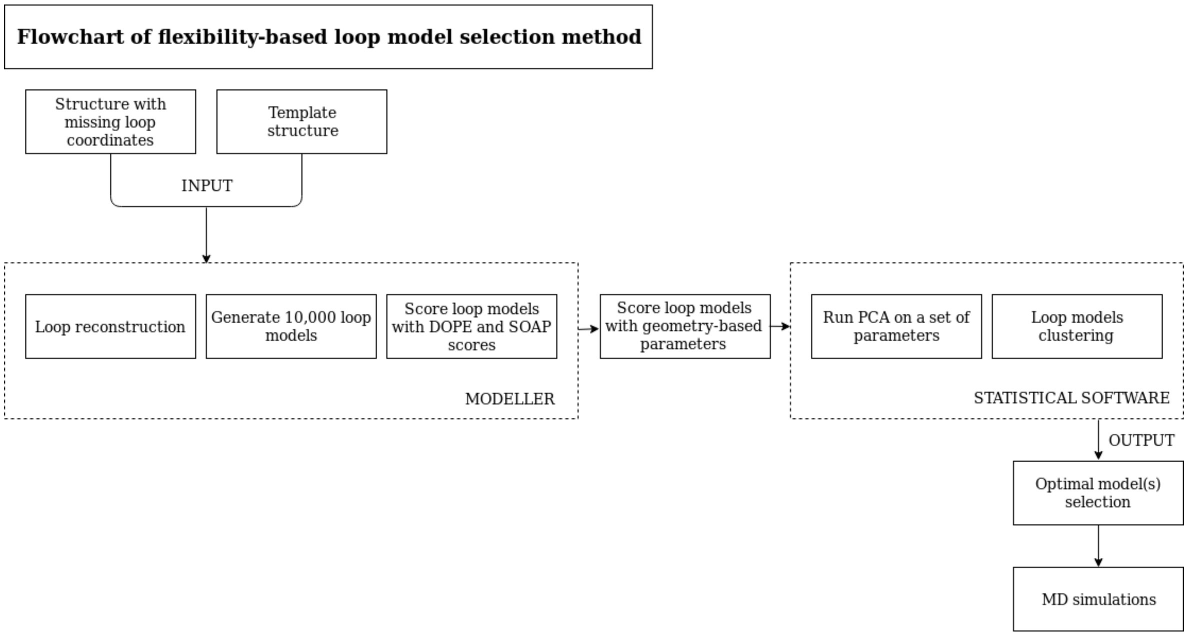

Figure S1. Flowchart of the loop reconstruction and model selection method. Abbreviations used: PCA – Principal Components Analysis; MD – molecular dynamics.

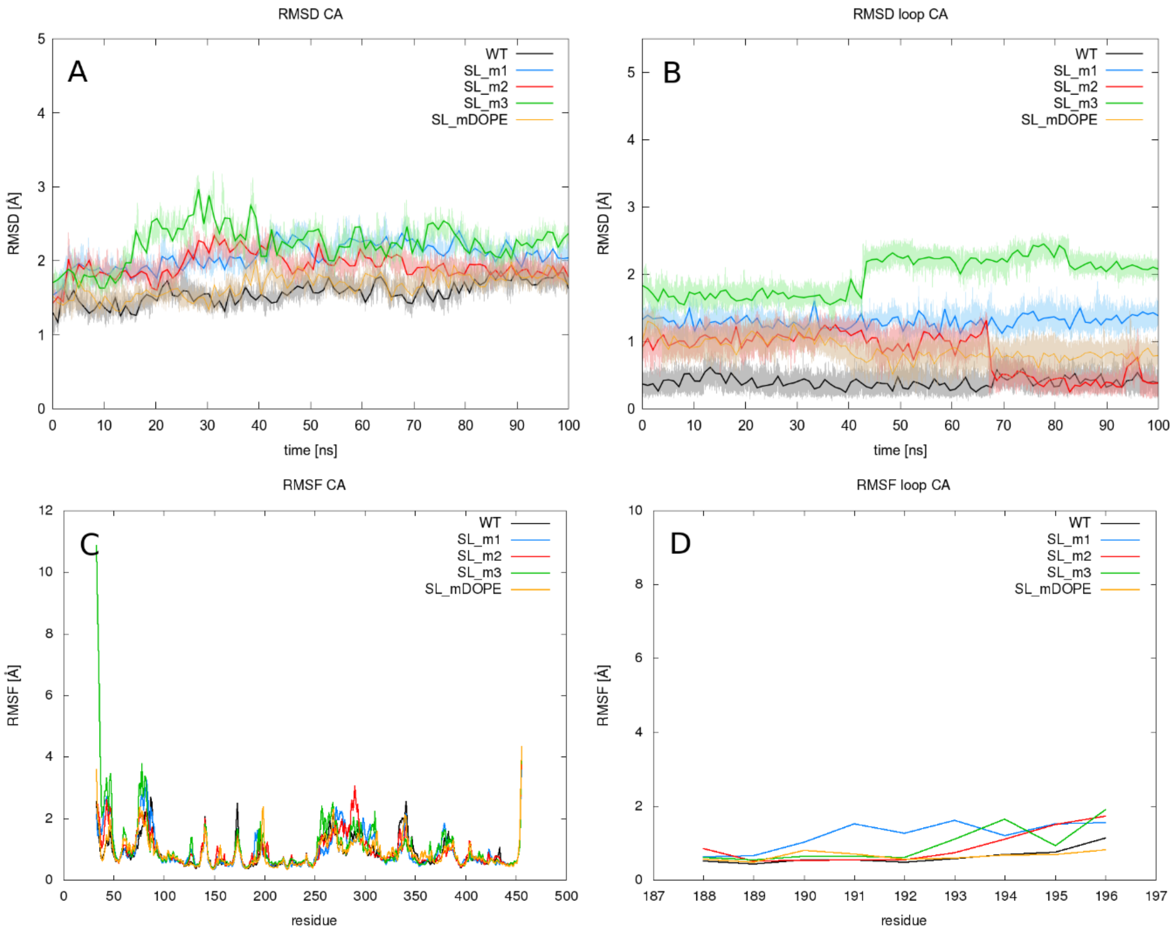

Figure S2. Plots of A) RMSD of backbone Cα atoms, B) RMSD of analyzed loop Cα atoms, C) RMSF of backbone Cα atoms, and D) RMSF of analyzed loop Cα atoms of a single MD run of each SL model.

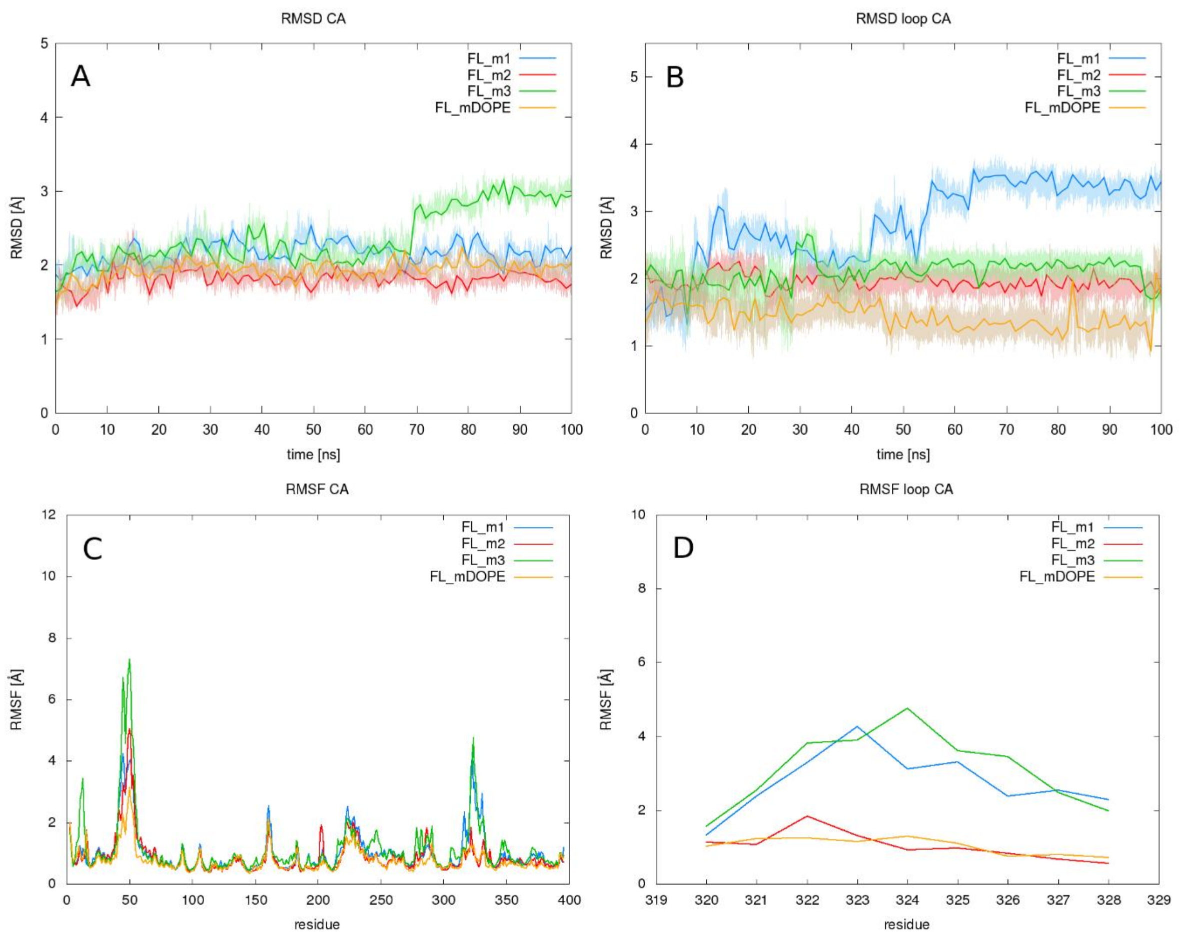

Figure S3. Plots of A) RMSD of backbone Cα atoms, B) RMSD of analyzed loop Cα atoms, C) RMSF of backbone Cα atoms, and D) RMSF of analyzed loop Cα atoms of a single MD run of each FL model.

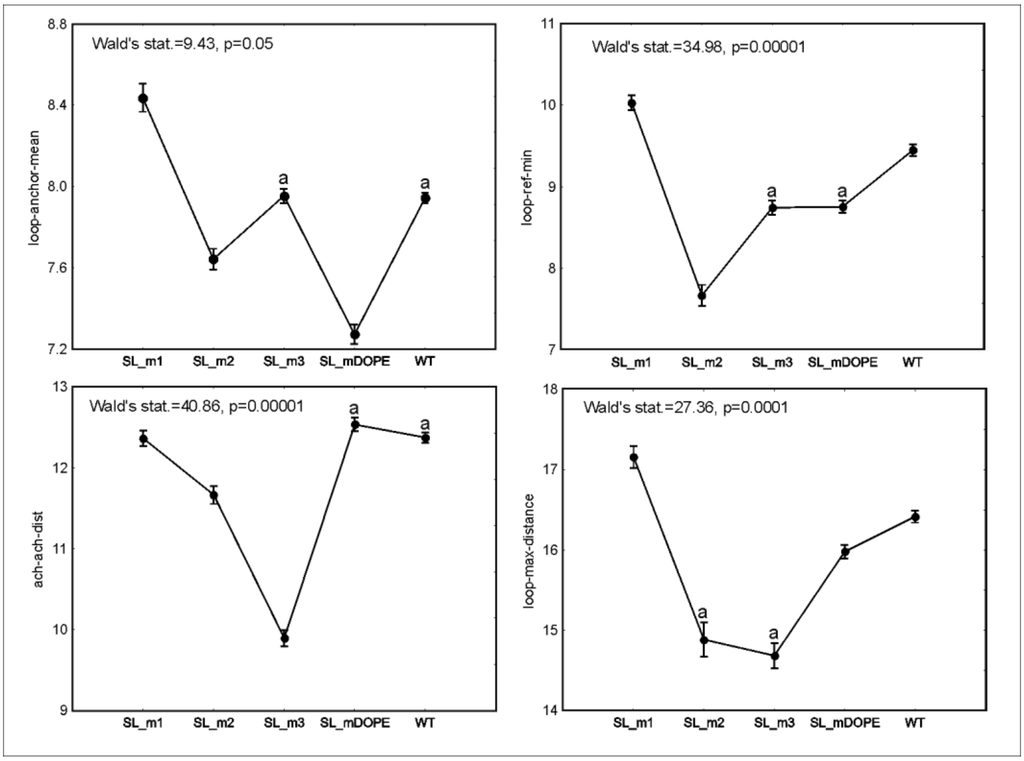

Figure S4. Mean parameters of the static loop models. Letters indicate no significant differences in mean values according to Tukey's HSD test.

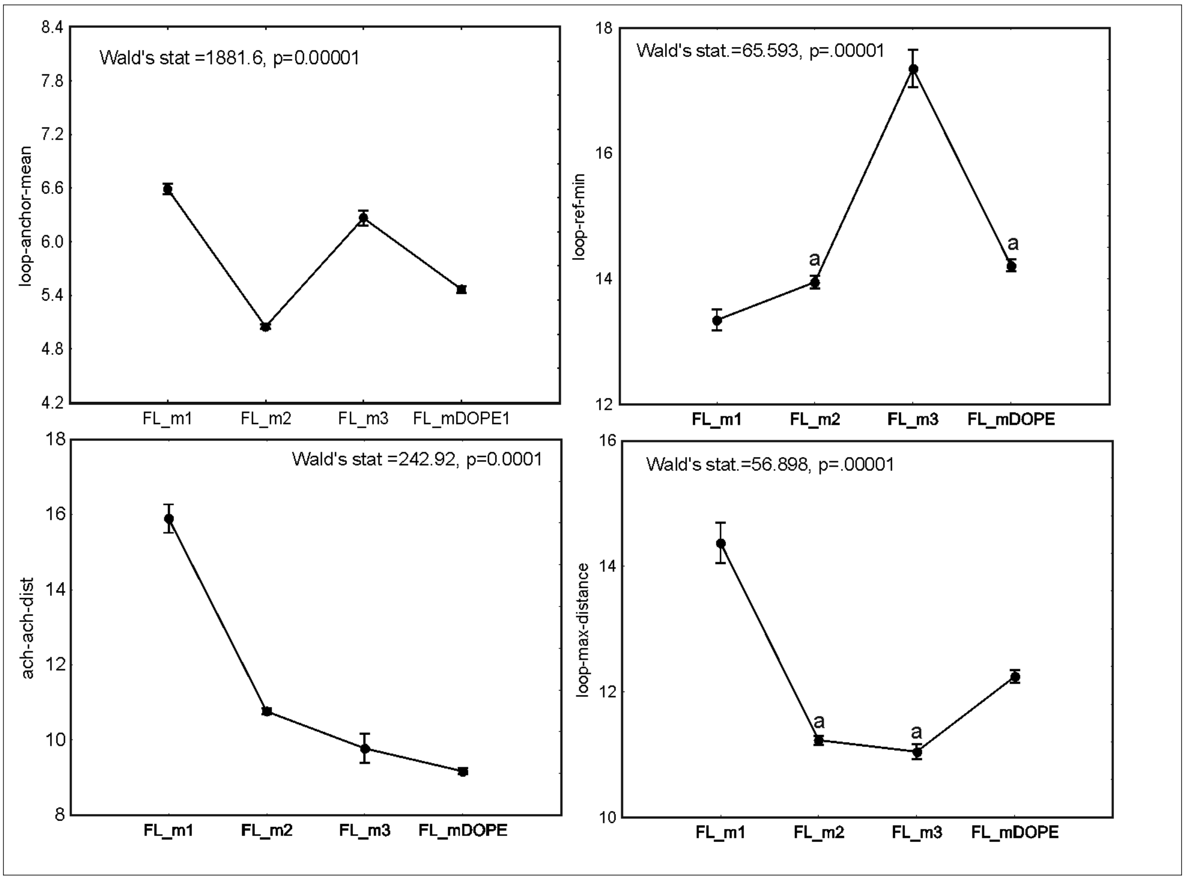

Figure S5. Mean parameters of the flexible loop models. Letters indicate no significant differences in mean values according to Tukey's HSD test.

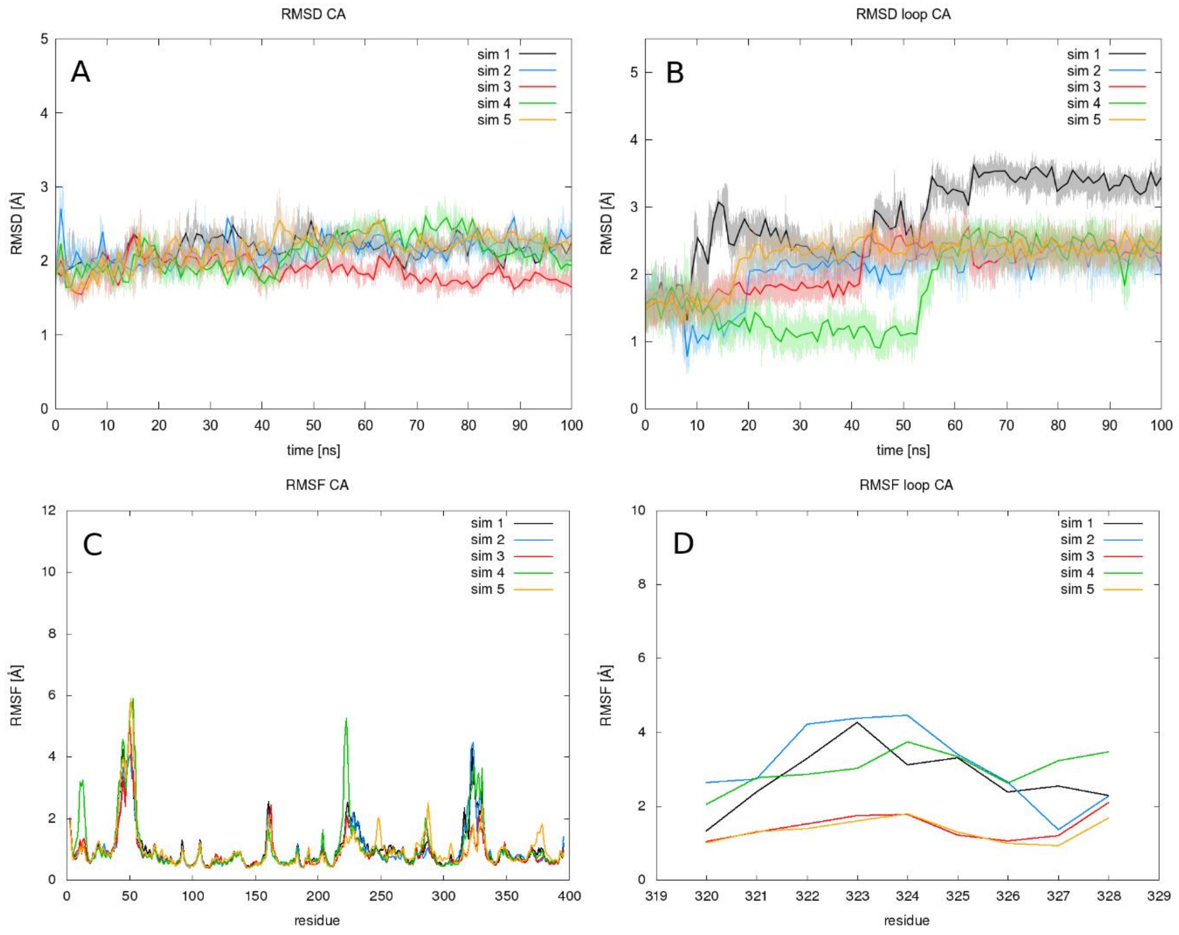

Figure S6. Plots of A) RMSD of backbone Cα atoms, B) RMSD of analyzed loop Cα atoms, C) RMSF of backbone Cα atoms, and D) RMSF of analyzed loop Cα atoms of five repetitions of a 100 ns MD run for the FL_m1 model.

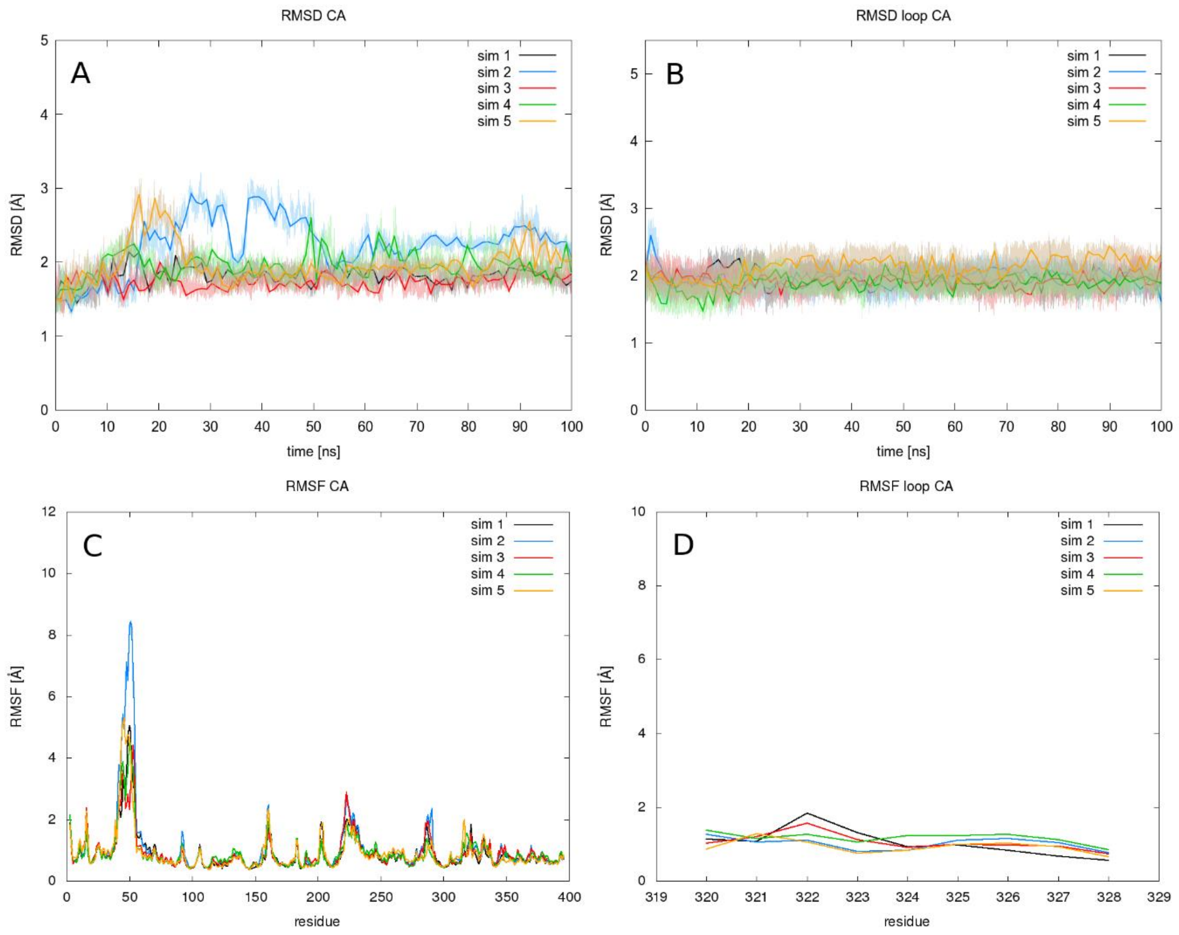

Figure S7. Plots of A) RMSD of backbone Cα atoms, B) RMSD of analyzed loop Cα atoms, C) RMSF of backbone Cα atoms, and D) RMSF of analyzed loop Cα atoms of five repetitions of a 100 ns MD run for the FL_m2 model.

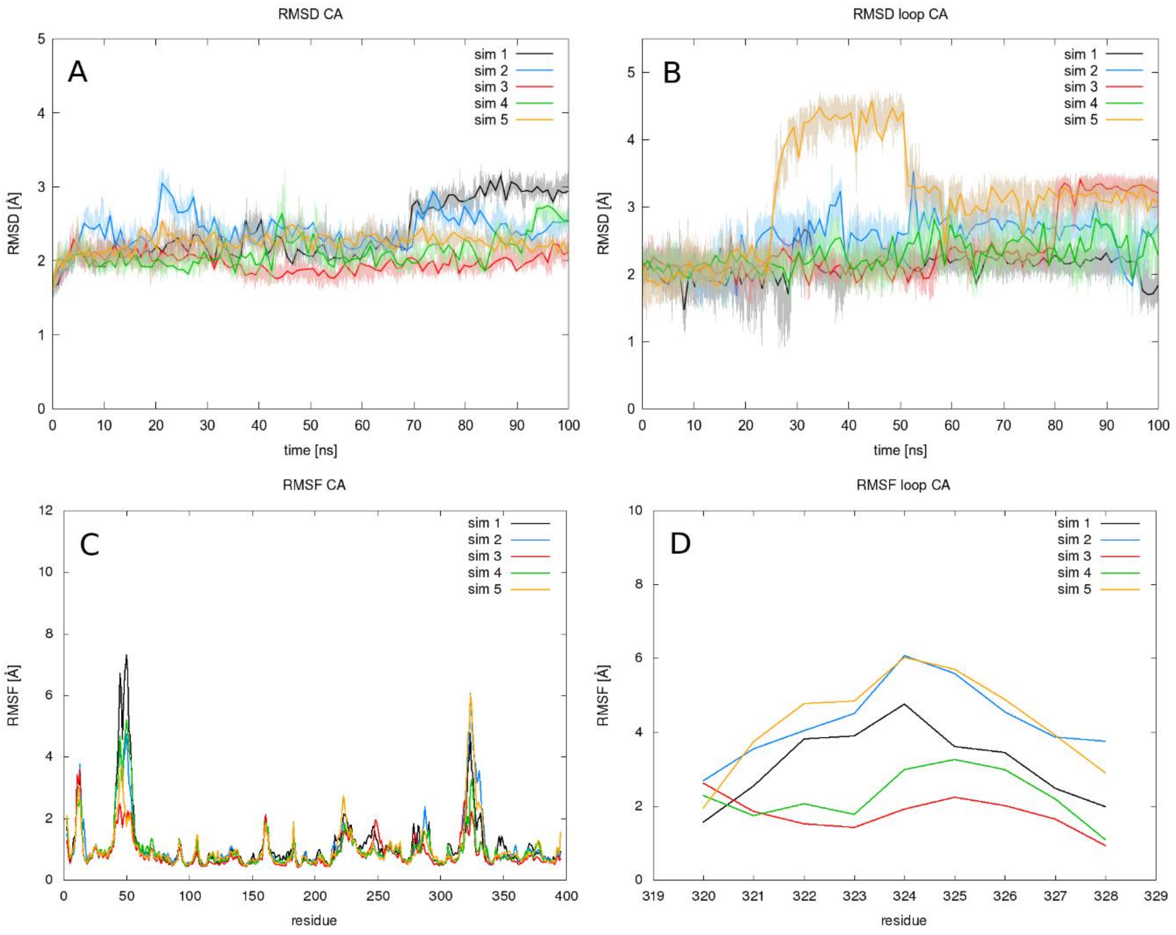

Figure S8. Plots of A) RMSD of backbone Cα atoms, B) RMSD of analyzed loop Cα atoms, C) RMSF of backbone Cα atoms, and D) RMSF of analyzed loop Cα atoms of five repetitions of a 100 ns MD run for the FL_m3 model.

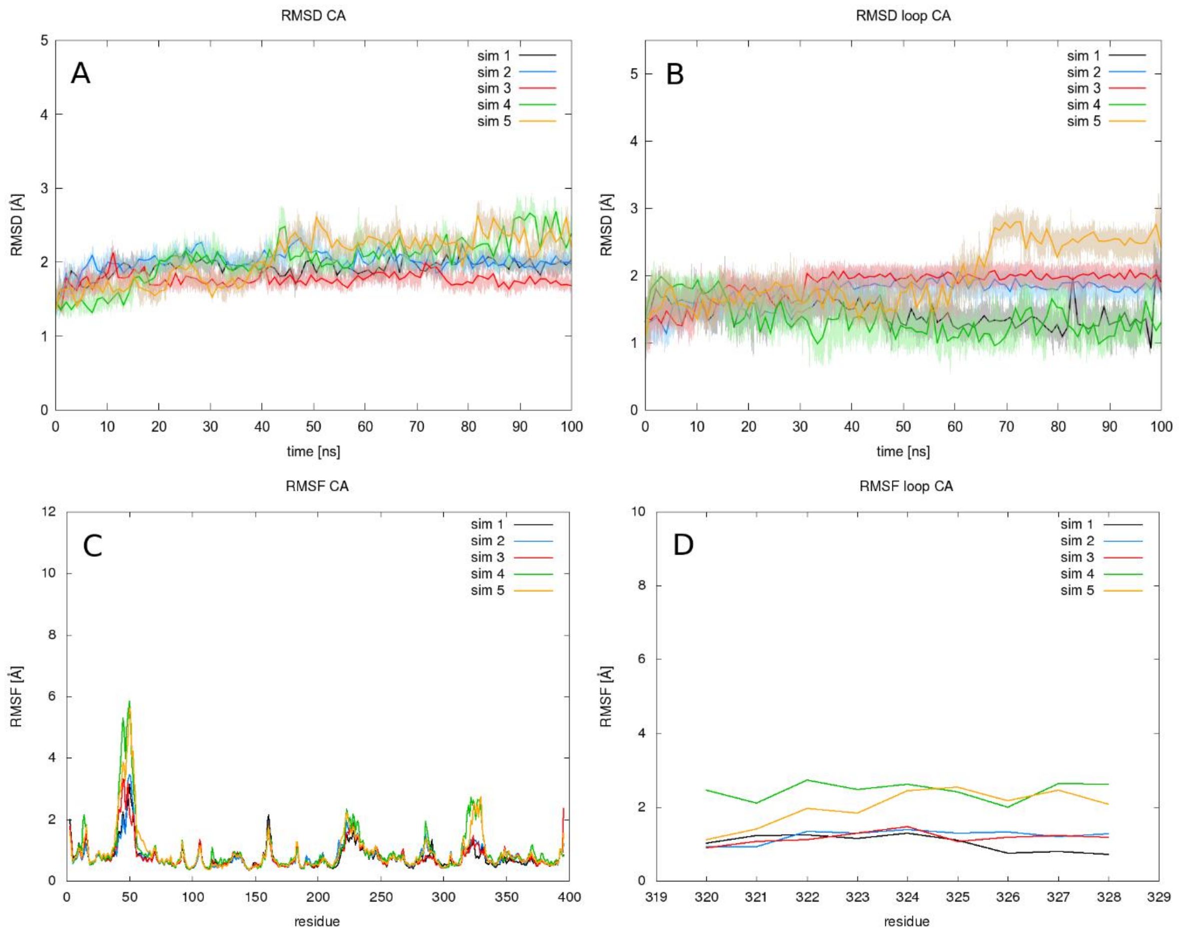

Figure S9. Plots of A) RMSD of backbone Cα atoms, B) RMSD of analyzed loop Cα atoms, C) RMSF of backbone Cα atoms, and D) RMSF of analyzed loop Cα atoms of five repetitions of a 100 ns MD run for the FL_mDOPE model.

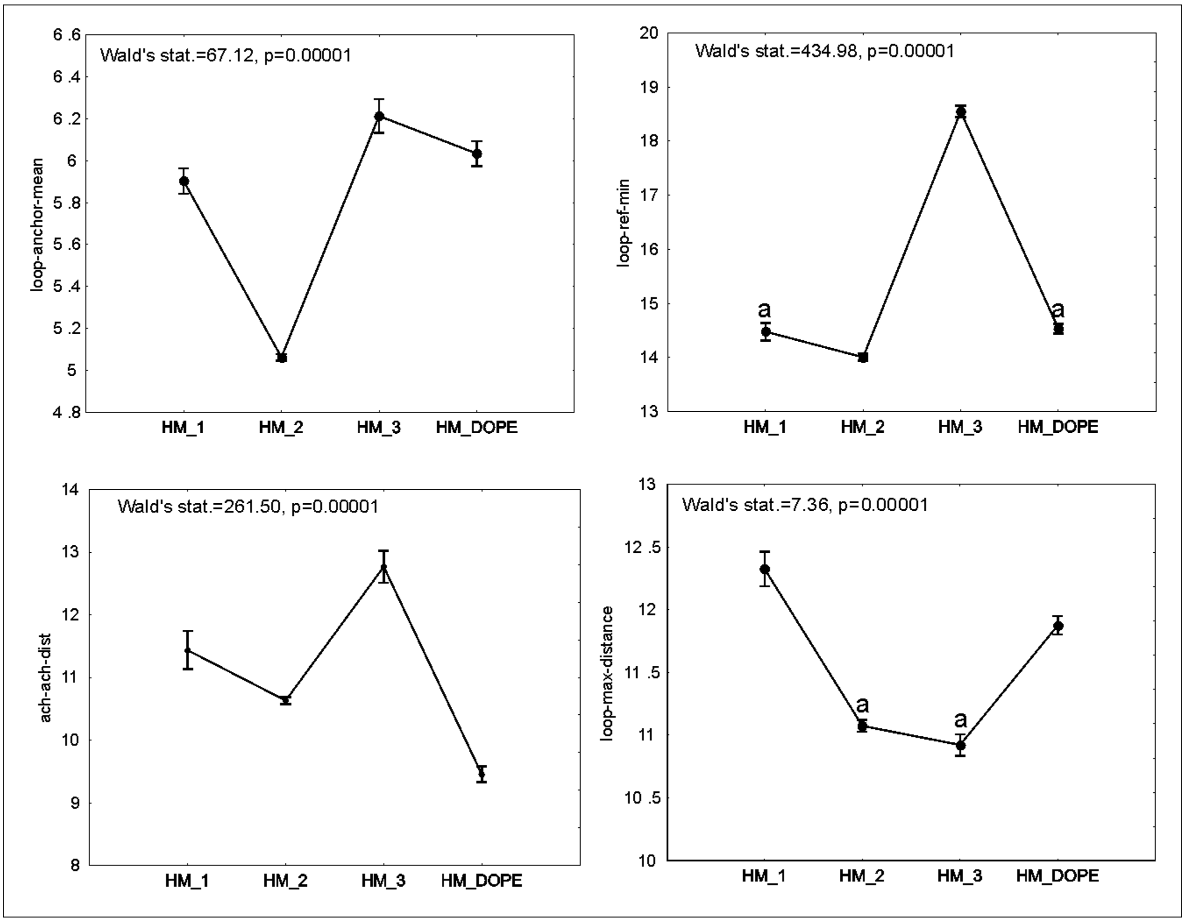

Figure S10. Mean parameters of the flexible loop models during 100 ns MD run with five repetitions. Letters indicate no significant differences in mean values according to Tukey's HSD test.

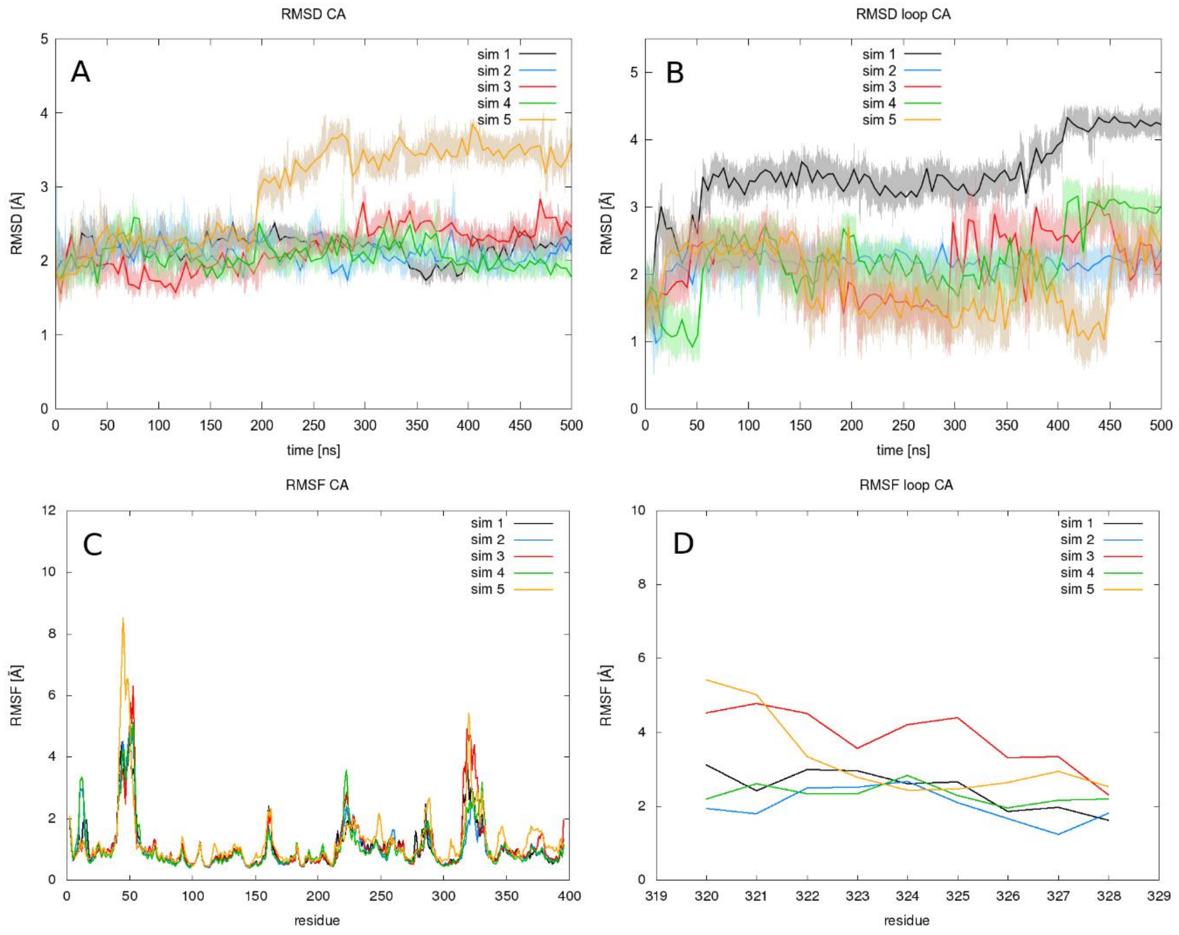

Figure S11. Plots of A) RMSD of backbone Cα atoms, B) RMSD of analyzed loop Cα atoms, C) RMSF of backbone Cα atoms, and D) RMSF of analyzed loop Cα atoms of five repetitions of a 500 ns MD run for the FL_m1 model.

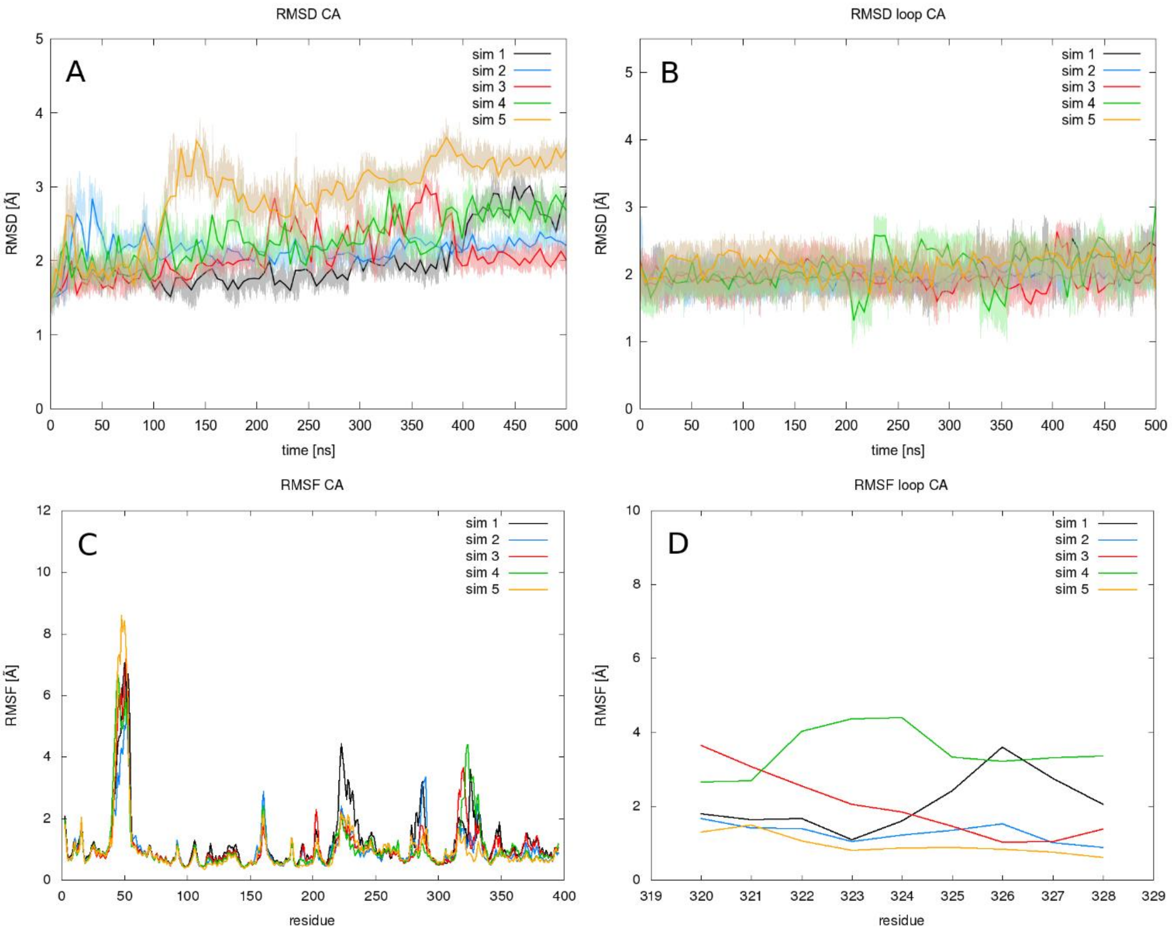

Figure S12. Plots of A) RMSD of backbone Cα atoms, B) RMSD of analyzed loop Cα atoms, C) RMSF of backbone Cα atoms, and D) RMSF of analyzed loop Cα atoms of five repetitions of a 500 ns MD run for the FL_m2 model.

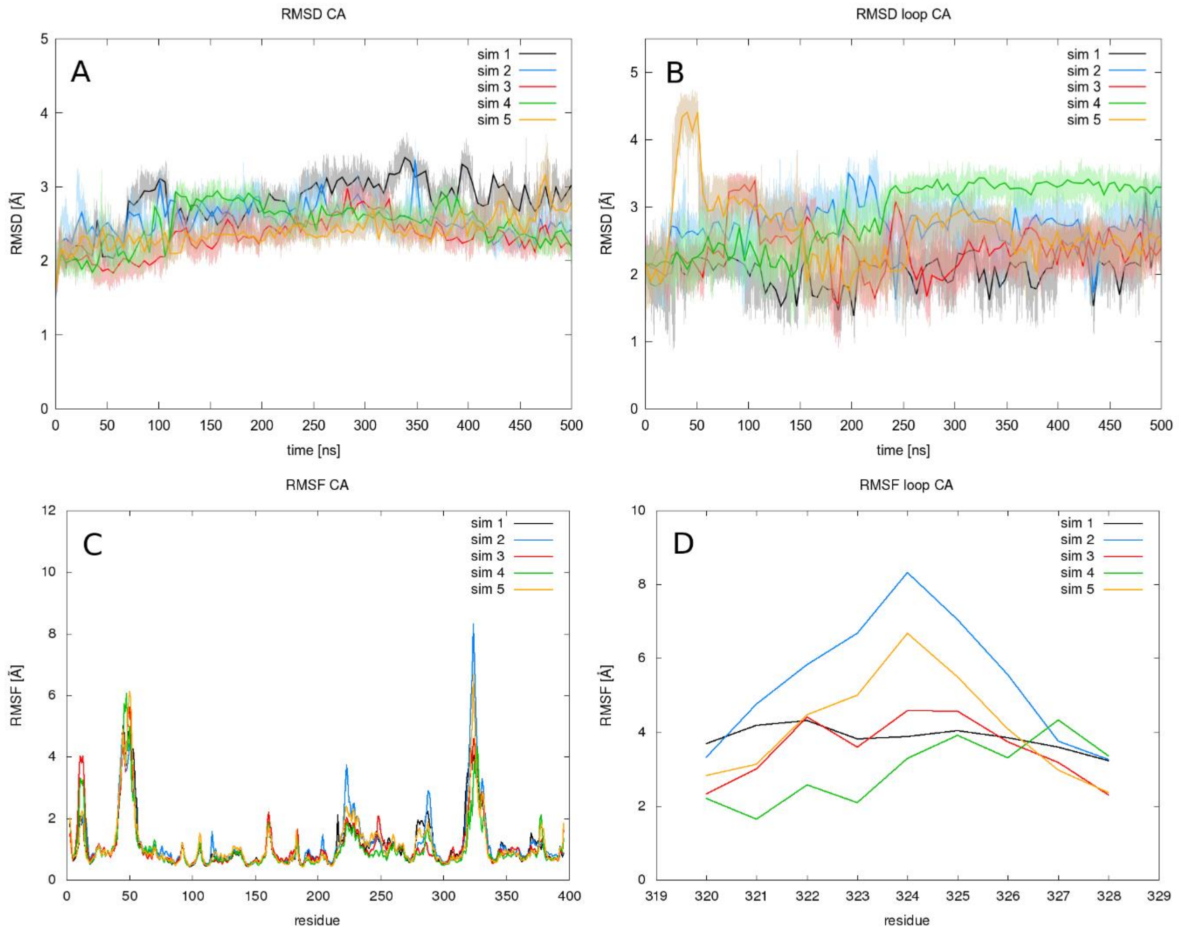

Figure S13. Plots of A) RMSD of backbone Cα atoms, B) RMSD of analyzed loop Cα atoms, C) RMSF of backbone Cα atoms, and D) RMSF of analyzed loop Cα atoms of five repetitions of a 500 ns MD run for the FL_m3 model.

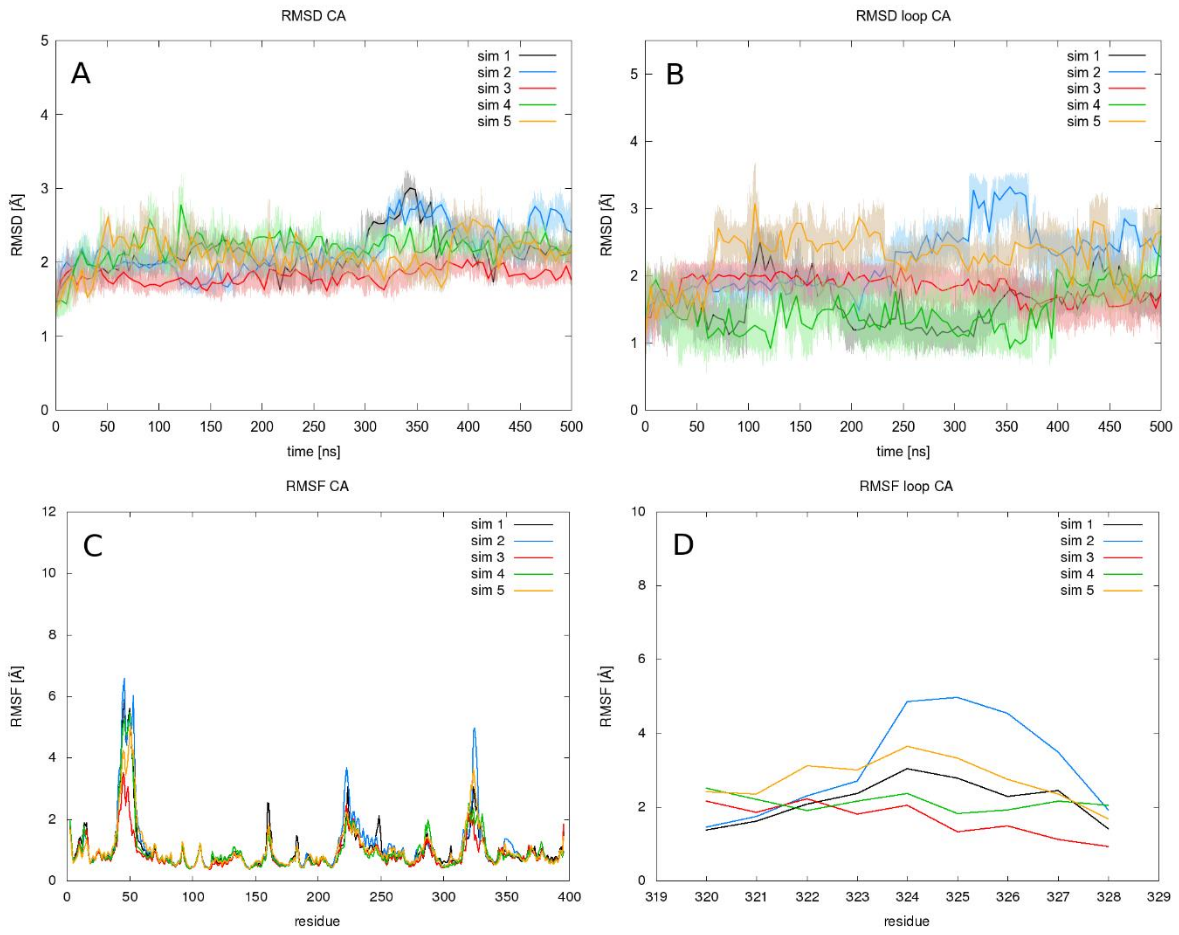

Figure S14. Plots of A) RMSD of backbone Cα atoms, B) RMSD of analyzed loop Cα atoms, C) RMSF of backbone Cα atoms, and D) RMSF of analyzed loop Cα atoms of five repetitions of a 500 ns MD run for the FL_mDOPE model.

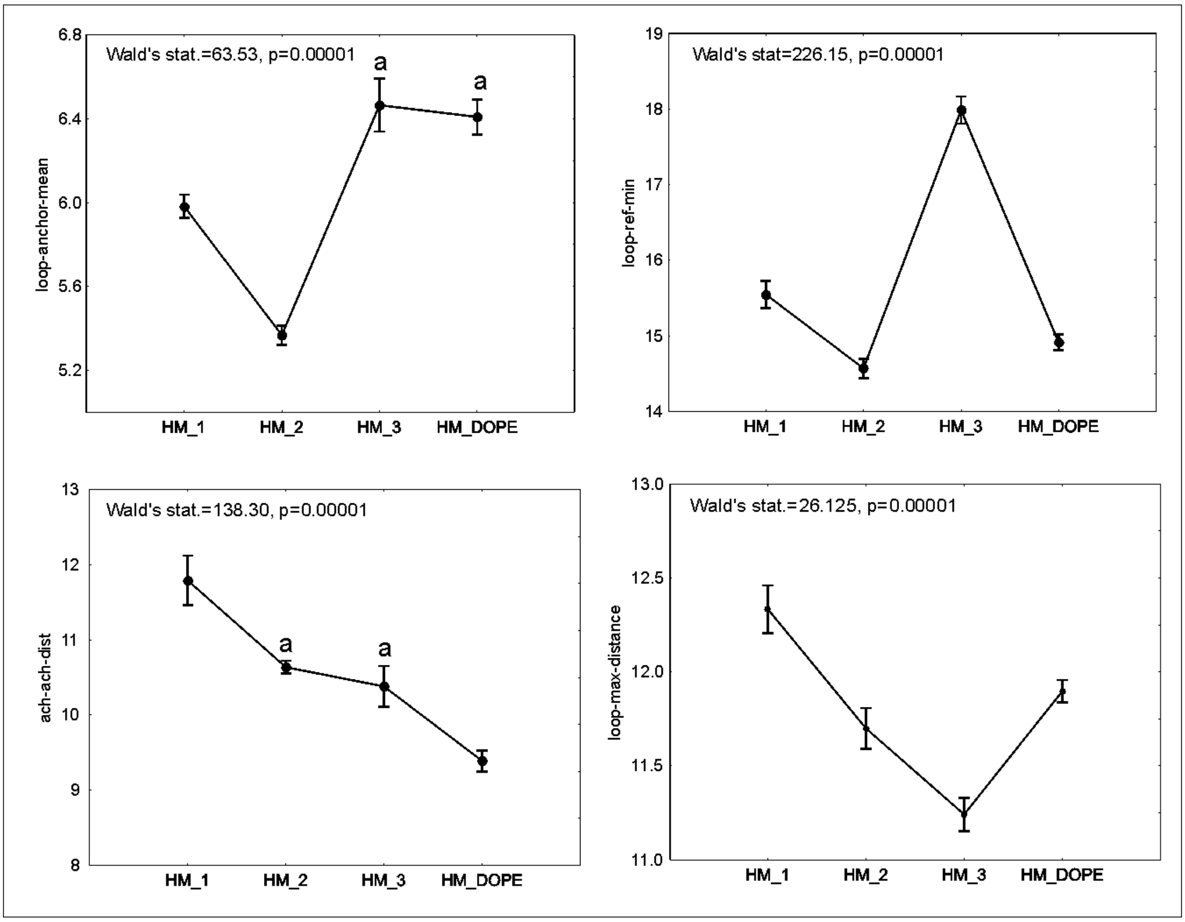

Figure S15. Mean parameters of the flexible loop models during 500 ns MD run in five repetitions. Letters indicate no significant differences in mean values according to Tukey's HSD test.

Table S1. The distribution of geometry-based parameters for FL and SL models sorted by the highest Relative Standard Deviation (RSD). The FL models have more variation in their parameters regarding loop's arch-shape than the SL models. Parameters with RSD > 8% are bolded.

FL models		SL models	
**parameter**	**RSD [%]**	**parameter**	**RSD [%]**
**loop-ref-min**	**11.2**	**ach-ach-dist**	**12.91**
**loop-max-distance**	**9.21**	loop-prot-v2a	4.55
**ach-ach-dist**	**8.52**	loop-max-distance	4.00
**loop-anchor-mean**	**8.02**	loop-anchor-mean	3.17
every-three-mean	4.81	every-two-mean	1.53
every-two-mean	3.52	loop-max-cons-distance-bb	1.42
loop-prot-v2a	3.24	every-three-mean	1.31
loop-max-cons-distance-bb	2.53	SOAP score	1.12
SOAP score	1.55	loop-prot-sh	1.02
loop-prot-sh	0.98	DOPE score	0.45
DOPE score	0.56	loop-ref-min	0.38

Table S2. The percentage ratio of identified loop states in 100 ns MD runs with particular FL models used as starting points. The most frequent conformation is in bold.

Loop state	FL_m1	FL_m2	FL_m3	FL_mDOPE
open	23.0%	1.6%	**48.6%**	7.2%
semi-open	30.4%	-	**48.0%**	28.2%
closed	**46.6%**	**98.4%**	3.4%	**64.6%**

Table S3. The percentage ratio of identified loop states in 500 ns MD runs with particular FL models used as starting points. The most frequent conformation is in bold.

Loop state	FL_m1	FL_m2	FL_m3	FL_mDOPE
open	27.6%	5.3%	**53%**	32.4%
semi-open	**45.3%**	19.5%	16.8%	15.0%
closed	27.1%	**75.2%**	30.2%	**52.6%**

Table S4. The GLM results showing the effect of extending simulations. The statistically significant loop parameters are highlighted gray.

		**degrees of freedom**	**Wald's Stat.**	**p**
loop-anchor-mean	Intercept	1	7151.623	**0.00001**
	sim	3	25.714	**0.00001**
loop-ref-min	Intercept	1	41945.39	**0.00001**
	sim	3	65.59	**0.00001**
ach-ach-dist	Intercept	1	25294.87	**0.00001**
	sim	3	242.92	**0.00001**
loop-max-distance	Intercept	1	30153.19	**0.00001**
	sim	3	56.9	**0.00001**
every-two-mean	Intercept	1	7551.509	**0.00001**
	sim	3	0.174	0.9816
every-three-mean	Intercept	1	11532.14	**0.00001**
	sim	3	0.65	0.8849
loop-max-cons-distance-bb	Intercept	1	136.1537	**0.00001**
	sim	3	0.0008	1.0000
loop-prot-sh	Intercept	1	41.79521	**0.00001**
	sim	3	0.0013	0.999988
loop-prot-v2a	Intercept	1	42.03813	**0.00001**
	sim	3	0.17446	0.9816
lnModDope	Intercept	1	24325.95	**0.00001**
	sim	3	0	1.0000
lnmodSoap	Intercept	1	34633.87	**0.00001**
	sim	3	0	1.0000
